# Sex Differences in Stress Susceptibility as a Key Mechanism Underlying Depression Risk

**DOI:** 10.1007/s11920-024-01490-8

**Published:** 2024-03-12

**Authors:** Summer Mengelkoch, George M. Slavich

**Affiliations:** Department of Psychiatry and Biobehavioral Sciences, University of California, Los Angeles, CA USA

**Keywords:** Stress susceptibility, Sex differences, Mechanisms, Depression, Intergenerational, Women’s health

## Abstract

**Purpose of Review:**

Although females are at relatively greater risk for a variety of disorders, including depression, the biological mechanisms underlying this striking health disparity remain unclear. To address this issue, we highlight sex differences in stress susceptibility as a key mechanism potentially driving this effect and describe the interacting inflammatory, hormonal, epigenomic, and social-environmental mechanisms involved.

**Recent Findings:**

Using the Social Signal Transduction Theory of Depression as a theoretical framework, women’s elevated risk for depression may stem from a tight link between life stress, inflammation, and depression in women. Further, research finds hormonal contraceptive use alters cortisol and inflammatory reactivity to acute stress in ways that may increase depression risk in females. Finally, beyond established epigenetic mechanisms, mothers may transfer risk for depression to their female offspring through stressful family environments, which influence stress generation and stress-related gene expression.

**Summary:**

Together, these findings provide initial, biologically plausible clues that may help explain the relatively greater risk for depression in females vs. males. Looking forward, much more research is needed to address the longstanding underrepresentation of females in biomedical research on the biology of stress and depression.

## Introduction

Experiencing major life stressors, such as a relationship breakup or persistent job insecurity, increases a person’s risk for developing numerous physical and mental health problems, including depression [1•, 2•]. However, despite similar rates of exposure to many stressor types, this risk is not shared equally by males and females, with females having much higher rates of depression compared to males following the pubertal transition. Indeed, the Centers for Disease Control and Prevention (CDC) recently reported that 57% of high-school aged females experienced persistent feelings of sadness or hopelessness in 2021, which is nearly twice the rates reported by their male peers [3]. These data are consistent with years of research showing that women are nearly twice as likely as men to develop depression following puberty [4].

Although men and women are approximately equally likely to experience most types of stressors, women more often face specific interpersonal life events that increase the risk for developing depression [5•]. The Social Signal Transduction Theory of Depression was the first theory to describe the full set of psychosocial and biological mechanisms through which interpersonal stressors, in particular, lead to depression [2•], and how sex differences in susceptibility to stress influence these processes [5•]. Here, we use the Social Signal Transduction Theory framework to discuss sex differences in susceptibility to stress and depression throughout the lifespan, along with the inflammatory, hormonal, and intergenerational mechanisms through which stress differently influences depression risk between the sexes. In doing so, we highlight the types of empirical studies needed to mechanistically understand how stressful experiences impact women differently than men, leading to their relatively greater risk of depression. We hope that elucidating mechanisms driving these associations will pave the way for novel therapeutic approaches that effectively target the stress-related mechanistic processes causing women to experience elevated rates of depression compared to men.

## Social Signal Transduction Theory of Depression

According to the Social Signal Transduction Theory of Depression, the human brain works closely with the immune system to keep the body safe from harm. It does this by surveying the external environment for potential threats and engaging protective psychological, biological, and behavioral systems to avoid or combat perceived threats. These threats vary in intensity and source and can be physical (i.e., threat of violence), social (i.e., exclusion), or environmental (i.e., resource scarcity). When a threat is detected, the body activates the sympathetic nervous system, hypothalamic–pituitary–adrenal (HPA) axis, and innate immune system, which results in elevated inflammatory activity, in addition to other biobehavioral effects. Although the activation of this stress response is highly adaptive and improves the odds of survival in the face of actual danger or injury, when stress responses are activated frequently or for prolonged periods of time, the activation can become health damaging [6, 7].

Consistent with the biological mechanisms proposed by the Social Signal Transduction Theory of Depression [2•, 5•], the body of evidence supporting a link between interpersonal stressors, inflammation, and depression has grown steadily over the years (e.g., [8•]). For example, in adolescent girls who are at high risk for psychopathology, greater interpersonal life stressor exposure predicted greater increases in depression over time, but only for those with a more pronounced proinflammatory response to an acute laboratory-based social stressor [9••]. Indeed, women, compared to men, are more likely to experience inflammation-induced shifts in mood and behavior, including greater feelings of social disconnection and loneliness when even transiently inflamed [10], which increases their risk of depression (for a review, see ref. [11]). In one study [10], researchers induced inflammation in the lab by administering an endotoxin to male and female participants. Endotoxin exposure increased inflammation, social disconnection, and depressed mood for both sexes; however, these effects were stronger for females compared to males. Although in general, women have been found to have an elevated susceptibility to social stressor-related depression, these effects depend, in part, on when stressors occur, the specific type of stressor experienced, and individual differences in the individual’s stress susceptibility, genetic predispositions, and the presence of psychosocial resources (e.g., optimism, social support) that can buffer one from the negative effects of stress.

## Sex Differences in Stress Susceptibility Across the Lifespan

Developmentally, early life stressor exposure has a significant impact on an individual’s stress reactivity and subsequent mental health outcomes, especially during two key developmental windows. First, stress begins to impact neural and HPA axis development in utero [12]. Maternal glucocorticoid levels influence the development of the fetus, often in sex-differentiated ways. For example, males exposed to high levels of maternal glucocorticoids have higher rates of attention deficit disorder and autism than females or males exposed to lower levels of maternal glucocorticoids [13]. However, females with elevated maternal glucocorticoid exposure have higher rates of anxiety and depression and exhibit elevated HPA axis reactivity later in life [14]. Although the effects of maternal stress are often observed in males during childhood, effects in females are more likely to emerge with the onset of puberty [15].

The second critical window of development during which time stressor exposure has an outsized impact on later health outcomes is from birth until about age six. During this time, exposure to major acute and especially chronic stressors promotes the development of a proinflammatory phenotype [16]. Although males are theorized to be more susceptible to their early life environmental conditions than females due to their developmental inflexibility [15], the inflammatory effects of early life stressor exposure persist more strongly in females throughout the lifespan [17, 18]. And, as alluded to above, one of the main mechanisms through which stress causes poor mental and physical health outcomes is inflammation [19•, 20].

## But *How*? Mechanisms Underlying Sex Differences in Stress Susceptibility

Given the role that inflammation plays in depression, sex differences in basal inflammatory activity—as well as inflammatory reactivity to social stressors—provide a biologically plausible explanation for women’s greater susceptibility to depression relative to men. Compared to men, women generally have higher basal inflammatory activity [21], with the proinflammatory properties of estradiol [21] likely driving this sex difference. The effects of sex steroid hormones on inflammation are complex and pleiotropic; however, estradiol has been found to increase risk for inflammation-related disorders and promote neuroinflammation in many concentrations [21–24] (see also ref. [5•] for more detailed discussion of biological mechanisms through which estradiol impacts inflammation and immune function).

Functionally, estradiol-induced elevated inflammation levels in women following puberty is theorized to be adaptive in that inflammation protects females and their offspring from pathogenic threats. However, elevated inflammatory activity also likely contributes to the greater prevalence of many female-dominated pathologies. For example, similar to rates of depression, women are also twice as likely as men to develop autoimmune diseases [25], most of which are marked by elevated inflammation. Sensitization of the immune system through chronic or repeated stressor exposure can contribute to autoimmunity, and associations between inflammation and autoimmunity are driven through several signaling pathways, including NF-κB and proinflammatory cytokine signaling [26–28]. That the proinflammatory properties of estradiol might influence depression risk also helps to explain why girls exhibit outcomes associated with early life stressor exposure during the pubertal transition, when estradiol levels rise and begin to fluctuate cyclically. Boys, on the other hand, tend to exhibit outcomes associated with early life stressor exposure earlier in life [15].

Beyond the impacts of elevated inflammation, pubertal-onset depression in girls is also influenced by psychosocial factors that shift during the pubertal transition, when girls face new societal expectations about their gender and sexuality, and neurobiological changes. For example, they may feel increasingly self-conscious and experience new types of social interactions. Girls who reach puberty prior to their peers are especially susceptible to stress-induced depression during this time [29], when they often feel isolated from social support networks and their peers. Further, elevated sex steroid hormone levels and cyclically changing sex steroid hormone levels during the pubertal transition are associated with neurobiological changes that may contribute to elevated depression rates in girls. Progesterone and its byproduct allopregnanolone alter GABA functioning [30, 31], whereas estradiol has been found to impact the serotonin system in ways which contribute to depressive symptoms [32, 33].

Sex steroid hormones also impact the HPA axis. Elevated estradiol levels are associated with a blunted cortisol response to stress, which itself can result in unchecked and elevated inflammatory activity in response to stressors, as cortisol typically serves an anti-inflammatory role in response to acute stress. However, we know strikingly little about the biological processes associated with acute and chronic stress responses in women and girls, as women and female animals were excluded from pre-clinical and clinical trials until the 1990s [34]. Around that time, Kirschbaum and colleagues [35•] made the groundbreaking discovery that men exhibit elevated cortisol reactivity following stress compared to naturally cycling women, who exhibit higher cortisol reactivity following stress compared to women using hormonal contraceptives. Although hormonal mechanisms were suggested, they were not assessed, nor were they followed up on.

More than 20 years later, researchers have yet to determine why or how sex differences in cortisol responses to acute stressors reliably occur, or how hormonal contraceptive use blunts cortisol reactivity. A recent review [36•] found that even today, females are underrepresented in human acute stress research, and that within studies that did include women, many did not investigate sex differences in stress-related processes or account for hormonal contraceptive use or menstrual cycle phase. This research gap has occurred even though these hormonal influences have been known to impact stress reactivity for the past 30 years (e.g., [35•, 37, 38, 39•, 40••, 41, 42]).

Given the lack of knowledge about female stress biology more generally, it is unsurprising that researchers know very little about the mechanisms through which at least some types of hormonal contraceptives impact HPA axis reactivity in at least some women. Hormonal contraceptives prevent pregnancy by delivering synthetic sex steroid hormones (i.e., progestins), which, unlike endogenous sex steroid hormones, are nonspecific in their binding affinity and bind with sex steroid hormone receptors, glucocorticoid receptors, and mineralocorticoid receptors alike. This binding promiscuity results in hormonal contraceptive use having a wide range of unanticipated side effects, which, in some women, include mood-related symptoms.

These effects have significant public health significance given that hormonal contraceptive use is widespread [43] and that most women in America use hormonal contraceptives for at least some period of their reproductive aged years [44]. Moreover, many women use hormonal contraceptives during adolescence, when unanticipated side effects may be stronger [45•, 46•]. Indeed, a population-based investigation using public health data in Denmark found that hormonal contraceptive users, but especially adolescent users, are more likely to develop depression compared to non-users [47]. Building on this finding, a recent study uncovered that doctors in Denmark with high rates of hormonal contraceptive prescriptions to adolescent girls have patients who subsequently develop depression at higher rates than do doctors with lower rates of hormonal contraceptive prescriptions to their adolescent patients [48••].

Although these studies indicate that hormonal contraceptive use is associated with elevated depression risk in women and that doctors’ prescribing habits may be contributing to this association, these studies were not designed to elucidate biological mechanisms through which hormonal contraceptives influence depression risk. However, recent empirical research has begun to explore these mechanisms by investigating associations between hormonal contraceptive use and inflammatory reactivity to the Trier Social Stress Test. In one recent study, for example, researchers found that levels of the key inflammatory cytokine interleukin-6 (IL-6) rose alongside cortisol in naturally cycling women; in women using hormonal contraceptives, however, the key inflammatory cytokine tumor necrosis factor-α (TNF-α) rose alongside cortisol, and rises in cortisol in this group were, in turn, associated with more negative affect in hormonal contraceptive users following psychosocial stress [40••]. This research suggests that hormonal contraceptive use may alter women’s stress reactivity in ways that make them less able to psychologically manage the stress they experience. Over time, these effects could elevate depression risk for at least some women using hormonal contraceptives. Given that there are many individual differences and moderating factors affecting biological mechanisms through which hormonal contraceptive use could increase women’s risk for depression, additional research is needed to better understand for which women hormonal contraceptive use increases depression risk and why. Such research could also explore hormonal contraceptive or non-hormonal contraceptive options that decrease, as opposed to increase, women’s risk of developing depression.

Beyond inflammatory and hormonal mechanisms that contribute to sex differences in stress susceptibility and depression risk, environmental factors influence these sex differences as well. For girls, more so than boys, having a depressed mother is a strong predictor of developing depression by age 20 [49]. Research seeking to understand how a mother’s depression risk is transferred to her daughter found that those with depression have a tendency to experience or generate more stressors in their lives (i.e., stress generation) [49]. This shared family environment, marked by frequent or severe stressors, in turn, has been found to predict daughters’ acute stressor exposure and depressive symptoms [50••].

Environmental conditions such as early life stressor exposure also influence the expression of key inflammatory (e.g., the conserved transcriptional response to adversity [51, 52•]) and HPA axis-related genes. Emerging research exploring the transcriptomic mechanisms through which chronic early life stress and mother’s depression history interact to influence the expression of these gene sets in adolescent girls has found that girls with depressed mothers exhibit dysregulated HPA axis-related gene expression patterns regardless of chronic stress exposure, a pattern also found in girls without depressed mothers but who have experienced chronic early life stress [53•]. These results suggest that maternal depression increases girls’ risk for dysregulated HPA axis gene expression, even in the absence of early life chronic stress exposure, highlighting one potential pathway through which maternal stress and depression can be passed from mother to daughter, beyond genetic contributions.

Although stressful family environments are one way stress and depression are passed down from one generation to the next, genetic and epigenetic factors that are also shared between mother and daughter further contribute to the intergenerational transfer of depression risk. For example, Meaney’s landmark research on stress-related epigenetic changes in rats demonstrated that chronic stressor exposure causes decreased maternal sensitivity and the methylation of stress-related genes, which can in turn affect both offspring behavior and stress reactivity [54]. Indeed, one way in which maternal stressor exposure has a stronger impact on female vs. male offspring depression risk is through the maintenance and exacerbation of these methylation patterns. Specifically, female fetuses have higher and more stress-reactive levels of DNA methyltransferase than male fetuses, which promotes the continuation of methylation of stress-related genes [55]. Despite advances in sequencing technologies that allow for sequencing of the genome, epigenome, and transcriptome, a straightforward mechanism for intergenerational transfer of stress and trauma has yet to be discovered [56, 57•].

## Additional Empirical Research Is Needed

**Fig. 1:**
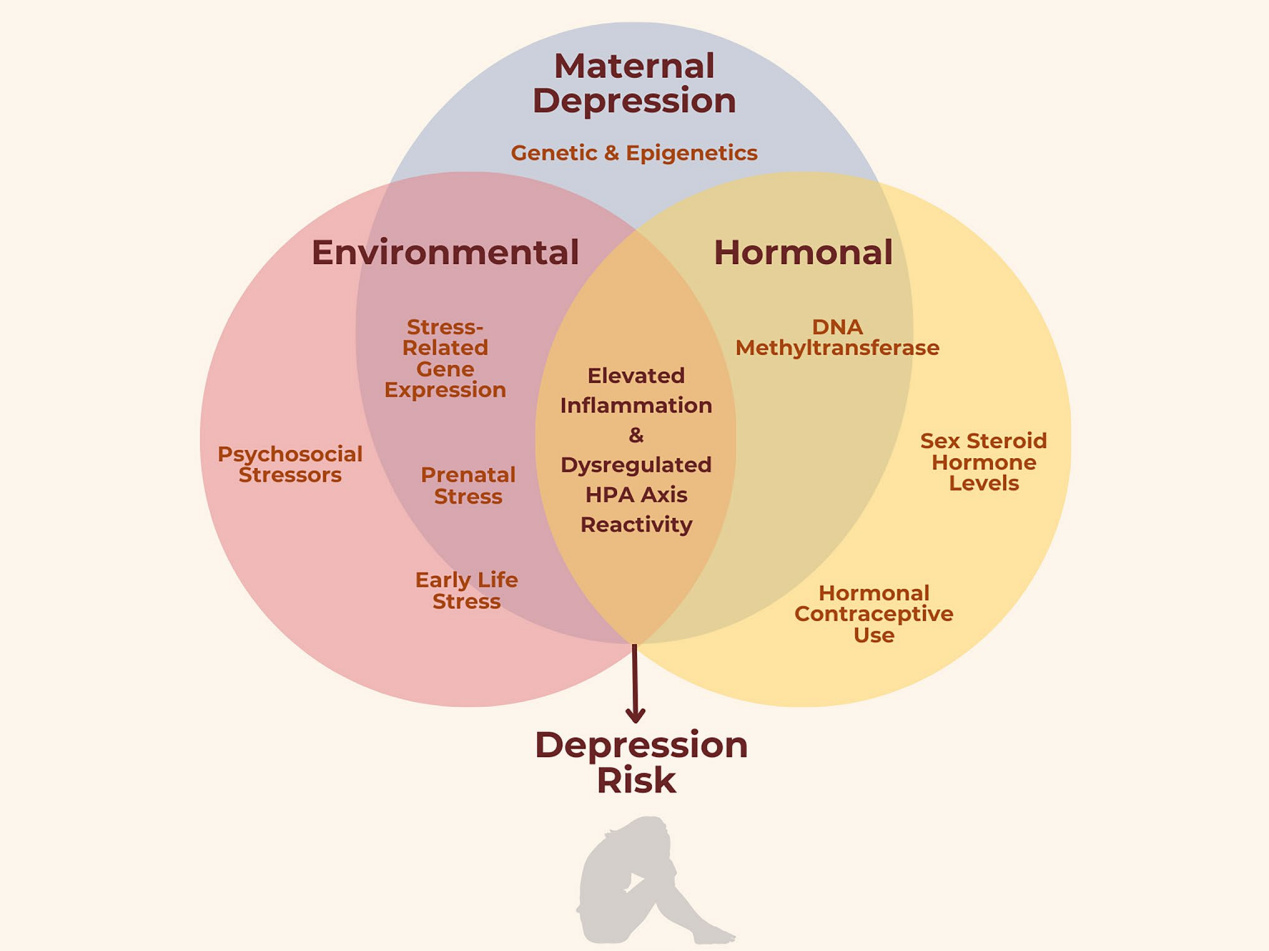
Overlapping mechanisms contributing to high rates of female depression. Maternal depression, environmental influences, and hormonal mechanisms interact with each other to contribute to elevated inflammation and dysregulated hypothalamic–pituitary–adrenal (HPA) axis reactivity, which, in turn, contribute to elevated rates of depression for women and girls compared to men and boys

In Fig. 1, we highlight some of the most important overlapping mechanisms contributing to high rates of depression in women and girls. To more fully understand sex differences in stress susceptibility and sex differences in depression risk, there are a few key empirical gaps that must be addressed. First, although the National Institutes of Health (NIH) now emphasizes the need to include women and female animals in clinical research [e.g., 57], decades of research on the topics reviewed herein have been conducted using only men and male animals. Although it is tempting to assume females and males only biologically differ in ways influenced by sex steroid hormone levels, or that female biology is just male biology with pregnancy and hormones added to the mix, such notions require empirical evidence and are unlikely to be true. As such, basic science research is needed that specifically assesses stress-related biological processes in women and female animals, and how these processes lead to both physical and psychological pathologies.

Moreover, although the inclusion of women and females in federally funded research has improved dramatically in the last 30 years, diseases that are female-dominated are underfunded relative to their disease burden compared to those that are male-dominated [58••] (see ref. [59•] for visualization). Basic, mechanistic research aimed at better understanding women’s stress-related biology is neither sexy nor very fundable at present, even though it will advance our understanding of health and disease for everyone. For example, understanding the female-specific processes in the etiology of depression will help to elucidate which causal factors, processes, or symptoms are characteristic of depression as a whole, characteristic of female depression, or characteristic of male depression. By understanding which processes are sex-specific, prevention and treatment efforts will ultimately become more efficacious for both sexes.

Further, researchers must be willing to design studies that can not only uncover sex differences and sex similarities but also elucidate mechanisms driving these differences and similarities. Indeed, many studies that assess both men and women do not test or report differences by biological sex. And when they do, it is often because sex was added to a hypothesized model as a covariate, a sex difference was found, and now sex differences are being reported as effects. While better than ignoring sex entirely, testing for sex differences as an afterthought, without careful study design, will not meaningfully move the field forward.

Studies that do not consider potential sex differences in study design run a few risks. First, there is the risk of imbalanced group sizes of males and females, which can decrease statistical power to detect true effects and increase the odds of erroneous effects being detected in small groups which are attributable to individual differences as opposed to sex differences. Second, if all participants are scheduled the same, without accounting for sex-specific factors such as cycle phase and hormonal contraceptive use, sex differences can be erroneously amplified or minimized. Instead, we need well-designed studies aimed at uncovering the mechanistic factors—whether they involve sex steroid hormones, gene expression, environmental factors, all of these (or other undiscussed mechanisms)—driving sex differences in depression risk following stress.

Researchers must exercise precision and attention to details when designing these studies, and the cumulation of results from many studies with different methodologies will be needed. Animal models are invaluable for carefully investigating potential biological mechanisms, although they do not always translate well to human models of depression. When using human participants, in turn, it is vital to ask participants to report their sex (male vs. female), as opposed to gender (man vs. woman), when investigating sex as a construct (although the intersecting effects of sex and gender are also in need of further investigation). It is also important to account for the many factors known to influence sex differences in stress reactivity and depression risk. For example, research with female participants should account for menstrual cycle phase and hormonal contraceptive use (see ref. [60] for recommendations), and given the lack of knowledge of causal associations, should use care when modeling these factors as covariates in the associations between sex, stress, and depression risk, in the event that these factors are actually more accurately modeled as mediators or colliders [61]. Measuring the impact of sex steroid hormones on stress-related processes is a key first step. However, the generation and testing of theoretical, biologically plausible pathways through which sex steroid hormone levels influence stress-related processes is also needed. Studies using multi-omics approaches that enable researchers to quantify tens of thousands of analytes from a single blood sample are well-suited for research into biological mechanisms driving sex differences in susceptibility to depression risk as well [62]. Further, research that uses the onset of hormonal contraceptive use as a natural experiment to better understand the impact of sex steroid hormones on stress reactivity and depression risk in an intensive longitudinal design will help to reveal mechanistic effects of hormonal contraceptive use and sex steroid hormones on mood and depression risk.

Finally, care must be taken by researchers when communicating results—both from basic science and applied research—as they pertain to sex differences in stress susceptibility. Ineffective science communication with eye-catching headlines (e.g., “Do hormones drive women’s votes?”; “UTSA prof suggests women vote with their vaginas” [63, 64]) can result in censorship of unfavorable findings, validation of sex-based discrimination, and slowed empirical progress. For example, research reporting that women’s sex steroid hormones or cycle phases influence their moods and behaviors, without appropriate context, contributes to the public stereotype that women and females are fickle or dominated by their hormonal states. This stereotype has been used to justify the exclusion of female animals from basic science research for decades. However, recent research has found that male mice actually exhibit more erratic behavior than do female mice, with estrus cycle phase having only a negligible effect on female behavior [65••]. Beyond careful science communication, careful research design will increase researchers’ confidence in their results, which can then be translated into novel therapeutic targets to treat and prevent depression, ideally in sex-specific ways.

## Conclusion

In conclusion, despite women experiencing nearly twice the rates of depression compared to men, we know far less about the psychobiological pathways through which psychopathology develops in females vs. males. Understanding potential sex-specific mechanisms through which women and girls are more likely to develop depression following stressful life experiences compared to men and boys will improve our understanding of the etiology of depression for both sexes and has the potential to improve our understanding of other stress-related diseases as well. Inflammatory processes, sex-steroid hormone and hormonal contraceptive effects on HPA axis reactivity to stress, and genomic and epigenomic processes—along with their interactions with social-environmental conditions—are likely biological mechanisms through which sex differences in stress susceptibility and increased risk for depression emerge. Precisely elucidating these mechanistic pathways will allow for more effective and targeted depression treatments, interventions, and prevention measures for those at high risk. We hope that this review inspires both basic science and applied research that uncovers targetable, female-specific, stress-related biological mechanisms through which life stressors are translated into elevated risk for depression in girls and women.

The time to improve our understanding of female biology and mental health is now, and doing so will help to greatly improve the lives of the roughly 50% of individuals on the planet who have been left behind by empirical research thus far.

## References

[1] Burani K, Brush CJ, Shields GS, Klein DN, Nelson B, Slavich GM, Hajcak G (2023). Cumulative lifetime acute stressor exposure interacts with reward responsiveness to predict longitudinal increases in depression severity in adolescence. Psychol Med.

[2] Slavich GM, Irwin MR (2014). From stress to inflammation and major depressive disorder: a Social Signal Transduction Theory of Depression. Psychol Bull.

[3] Centers for Disease Control and Prevention (CDC). Youth risk behavior survey data summary & trends report. 2023;2011–21.

[4] Salk RH, Hyde JS, Abramson LY (2017). Gender differences in depression in representative national samples: meta-analyses of diagnoses and symptoms. Psychol Bull.

[5] Slavich GM, Sacher J (2019). Stress, sex hormones, inflammation, and major depressive disorder: extending Social Signal Transduction Theory of Depression to account for sex differences in mood disorders. Psychopharmacology.

[6] Slavich GM, Harkness KL, Hayden EP (2020). Psychoneuroimmunology of stress and mental health. The Oxford handbook of stress and mental health.

[7] Slavich GM, Auerbach RP. Stress and its sequelae: depression, suicide, inflammation, and physical illness. In: Butcher JN, Hooley JM (eds) APA handbook of psychopathology: psychopathology: understanding, assessing, and treating adult mental disorders. Am Psychol Assoc, Washington. 2018;1:375–40.

[8] Madison AA, Andridge R, Shrout MR, Renna ME, Bennett JM, Jaremka LM (2022). Frequent interpersonal stress and inflammatory reactivity predict depressive-symptom increases: two tests of the Social-Signal-Transduction Theory of Depression. Psychol Sci.

[9] Slavich GM, Giletta M, Helms SW, Hastings PD, Rudolph KD, Nock MK, Prinstein MJ (2020). Interpersonal life stress, inflammation, and depression in adolescence: testing Social Signal Transduction Theory of Depression. Depress Anxiety.

[10] Moieni M, Irwin MR, Jevtic I, Olmstead R, Breen EC, Eisenberger NI (2015). Sex differences in depressive and socioemotional responses to an inflammatory challenge: implications for sex differences in depression. Neuropsychopharmacol.

[11] Derry HM, Padin AC, Kuo JL, Hughes S, Kiecolt-Glaser JK (2015). Sex differences in depression: does inflammation play a role?. Curr Psychiatry Rep.

[12] Sutherland S, Brunwasser SM (2018). Sex differences in vulnerability to prenatal stress: a review of the recent literature. Curr Psychiatry Rep.

[13] Shao S, Wang J, Huang K, Wang S, Liu H, Wan S, Yan S, Hao J, Zhu P, Tao F (2020). Prenatal pregnancy-related anxiety predicts boys’ ADHD symptoms via placental C-reactive protein. Psychoneuroendocrinology.

[14] Kim D-J, Davis EP, Sandman CA, Sporns O, O’Donnell BF, Buss C, Hetrick WP (2017). Prenatal maternal cortisol has sex-specific associations with child brain network properties. Cereb Cortex.

[15] Hodes GE, Epperson CN (2019). Sex differences in vulnerability and resilience to stress across the life span. Biol Psychiat.

[16] Miller GE, Chen E (2010). Harsh family climate in early life presages the emergence of a proinflammatory phenotype in adolescence. Psychol Sci.

[17] Baldwin JR, Arseneault L, Caspi A (2018). Childhood victimization and inflammation in young adulthood: a genetically sensitive cohort study. Brain Behav Immun.

[18] Kim S, Watt T, Ceballos N, Sharma S (2019). Adverse childhood experiences and neuroinflammatory biomarkers—the role of sex. Stress Health.

[19] Furman D, Campisi J, Verdin E (2019). Chronic inflammation in the etiology of disease across the life span. Nat Med.

[20] Slavich GM (2015). Understanding inflammation, its regulation, and relevance for health: a top scientific and public priority. Brain Behav Immun.

[21] Klein SL, Flanagan KL (2016). Sex differences in immune responses. Nat Rev Immunol.

[22] Klein SL (2000). The effects of hormones on sex differences in infection: from genes to behavior. Neurosci Biobehav Rev.

[23] Villa A, Vegeto E, Poletti A, Maggi A (2016). Estrogens, neuroinflammation, and neurodegeneration. Endocr Rev.

[24] Gilliver SC (2010). Sex steroids as inflammatory regulators. J Steroid Biochem Mol Biol.

[25] Hayter SM, Cook MC (2012). Updated assessment of the prevalence, spectrum and case definition of autoimmune disease. Autoimmun Rev.

[26] Barnabei L, Laplantine E, Mbongo W, Rieux-Laucat F, Weil R. NF-κB: at the borders of autoimmunity and inflammation. Front Immunol. 2021;12. 10.3389/fimmu.2021.716469 PMC8381650 34434197

[27] Neurath MF, Finotto S (2011). IL-6 signaling in autoimmunity, chronic inflammation and inflammation-associated cancer. Cytokine Growth Factor Rev.

[28] Sharif K, Watad A, Coplan L, Lichtbroun B, Krosser A, Lichtbroun M (2018). The role of stress in the mosaic of autoimmunity: an overlooked association. Autoimmun Rev.

[29] Ge X, Conger RD, Elder GH (2001). Pubertal transition, stressful life events, and the emergence of gender differences in adolescent depressive symptoms. Dev Psychol.

[30] Burke CS, Susser LC, Hermann AD (2019). GABAA dysregulation as an explanatory model for late-onset postpartum depression associated with weaning and resumption of menstruation. Arch Womens Ment Health.

[31] Smith SS (2013). The influence of stress at puberty on mood and learning: role of the α4βδ GABAA receptor. Neuroscience.

[32] Frokjaer VG, Pinborg A, Holst KK, Overgaard A, Henningsson S, Heede M (2015). Role of serotonin transporter changes in depressive responses to sex-steroid hormone manipulation: a positron emission tomography study. Biol Psychiat.

[33] Mehta D, Rex-Haffner M, Søndergaard HB, Pinborg A, Binder EB, Frokjaer VG (2019). Evidence for oestrogen sensitivity in perinatal depression: pharmacological sex hormone manipulation study. Br J Psychiatry.

[34] Nadel MV. (Testimony) problems in implementing policy on women in study populations, Subcommittee on Housing and Consumer Interest and Select Committee on Aging, House of Representatives, 101^st^ Congress. 1990.

[35] Kirschbaum C, Kudielka BM, Gaab J, Schommer NC, Hellhammer DH (1999). Impact of gender, menstrual cycle phase, and oral contraceptives on the activity of the hypothalamus-pituitary-adrenal axis. Psychosom Med.

[36] Rrapaj A, Landau AM, Winterdahl M (2023). Exploration of possible sex bias in acute social stress research: a semi-systematic review. Acta Neuropsychiatr.

[37] Larsen B, Cox A, Colbey C, Drew M, McGuire H, Fazekas de St Groth B, et al. Inflammation and oral contraceptive use in female athletes before the Rio Olympic games. Front Physiol. 2020;11:497. 10.3389/fphys.2020.00497 PMC7261912 32523546

[38] Lovallo WR, Cohoon AJ, Acheson A, Vincent AS, Sorocco KH (2019). Cortisol stress reactivity in women, diurnal variations, and hormonal contraceptives: studies from the Family Health Patterns Project. Stress.

[39] Masama C, Jarkas DA, Thaw E, Daneshmend AZB, Franklyn SI, Beaurepaire C, McQuaid RJ (2022). Hormone contraceptive use in young women: altered mood states, neuroendocrine and inflammatory biomarkers. Horm Behav.

[40] Mengelkoch S, Gassen J, Slavich GM, Hill SE (2023). Hormonal contraceptive use is associated with differences in women’s inflammatory and psychological reactivity to an acute social stressor. Brain Behav Immun.

[41] Nielsen SE, Segal SK, Worden IV, Yim IS, Cahill L (2013). Hormonal contraception use alters stress responses and emotional memory. Biol Psychol.

[42] Roche DJO, King AC, Cohoon AJ, Lovallo WR (2013). Hormonal contraceptive use diminishes salivary cortisol response to psychosocial stress and naltrexone in healthy women. Pharmacol Biochem Behav.

[43] United Nations, Department of Economic and Social Affairs, Population Division. Contraceptive use by method 2019: data booklet (ST/ESA/SER.A/435). 2019.

[44] Daniels K. Contraceptive methods women have ever used: United States, 1982–2010. U.S. Department of Health and Human Services, Centers for Disease Control and Prevention, National Center for Health Statistics. 2013.

[45] Anderl C, Li G, Chen FS (2020). Oral contraceptive use in adolescence predicts lasting vulnerability to depression in adulthood. Child Psychol Psychiatry.

[46] Sharma R, Smith SA, Boukina N (2020). Use of the birth control pill affects stress reactivity and brain structure and function. Horm Behav.

[47] Skovlund CW, Mørch LS, Kessing LV, Lidegaard Ø (2016). Association of hormonal contraception with depression. JAMA Psychiat.

[48] •• Costa-Ramón A, Daysal NM, Rodriguez-Gonzalez A. The oral contraceptive pill and adolescents’ mental health. 2023 (preprint). **In Denmark, the likelihood of a depression diagnosis and antidepressant use increased shortly after hormonal contraceptive initiation. Further, being assigned to a high prescribing physician strongly predicted hormonal contraceptive use by age 16 and lead to worse mental health outcomes between ages 16 and 18. Because this was a population level study using health records, these results are not caveated by typical concerns of empirical research on hormonal contraceptive side effects, such as survivorship bias or lack of random assignment (e.g., self-selection).**

[49] Hammen C (2018). Risk factors for depression: an autobiographical review. Annu Rev Clin Psychol.

[50] Murphy MLM, Sichko S, Bui TQ, Libowitz MR, Shields GS, Slavich GM (2023). Intergenerational transmission of lifetime stressor exposure in adolescent girls at differential maternal risk for depression. J Clin Psychol.

[51] Slavich GM, Cole SW (2013). The emerging field of human social genomics. Clin Psychol Sci.

[52] Slavich GM, Mengelkoch S, Cole SW (2023). Human social genomics: concepts, mechanisms, and implications for health. Lifestyle Medicine.

[53] • Mengelkoch S, Alley JC, Cole SW, Slavich GM (*in preparation*) Genomic evidence of HPA axis dysregulation in adolescent girls at risk for depression. **Adolescent girls with depressed mothers exhibited dysregulated gene expression patterns (i.e., FKBP5 and NR3C1), regardless of chronic life stress experiences, similar to patterns of dysregulation found in girls without depressed mothers who have chronic life stress experiences. These results suggest that intergenerational transfer of depression risk for girls may have genomic underpinnings.**

[54] Meaney MJ, Szyf M, Seckl JR (2007). Epigenetic mechanisms of perinatal programming of hypothalamic-pituitary-adrenal function and health. Trends Mol Med.

[55] Mueller BR, Bale TL (2008). Sex-specific programming of offspring emotionality after stress early in pregnancy. J Neurosci.

[56] Bowers ME, Yehuda R, Chen A (2020). Chapter 17 - intergenerational transmission of stress vulnerability and resilience. Stress resilience.

[57] NIH Guide: NIH guidelines on the inclusion of women and minorities as subjects in clinical research. 1994;23(11). 11567286

[58] •• Mirin AA. Gender disparity in the funding of diseases by the U.S. National Institutes of Health. Journal of Women’s Health. 2021;30(7):956–63. **National Institutes of Health applies a disproportionate share of its resources toward diseases that affect primarily men, at the expense of those that affect primarily women.** 10.1089/jwh.2020.8682 PMC8290307 33232627

[59] • Smith K. The funding gender gap Nature 2023;617(7959):28-9. **A visualization of the disconnect between disease burden and National Institutes of Health funding of female- and male-dominated diseases. For an outstanding dynamic visualization, see **https://www.nature.com/immersive/d41586-023-01475-2/index.html

[60] Hill SE, Mengelkoch S (2023). Moving beyond the mean: promising research pathways to support a precision medicine approach to hormonal contraception. Front Neuroendocrinol.

[61] Moriarity DP, Mengelkoch S, Slavich GM (2023). Incorporating causal inference perspectives into psychoneuroimmunology: a simulation study highlighting concerns about controlling for adiposity in immunopsychiatry. Brain Behav Immun.

[62] Mengelkoch S, Schüssler-Fiorenza Rose SM, Lautman Z, Alley JC, Roos LG, Ehlert B (2023). Multi-omics approaches in psychoneuroimmunology and health research: conceptual considerations and methodological recommendations. Brain Behav Immun.

[63] Larson L. CNN pulls story “Do hormones drive women’s votes?” story after Twitter backlash. In: Mail Online. https://www.dailymail.co.uk/news/article-2223271/CNN-pulls-story-Do-hormones-drive-womens-votes-story-Twitter-backlash.html. 2012. Accessed 5 Jan 2024.

[64] Daily S. UTSA prof suggests women vote with their vaginas. In: San Antonio Current. https://www.sacurrent.com/news/utsa-prof-suggests-women-vote-with-their-vaginas-2252577. 2012. Accessed 5 Jan 2024.

[65] Levy DR, Hunter N, Lin S, Robinson EM, Gillis W, Conlin EB, Anyoha R, Shansky RM, Datta SR (2023). Mouse spontaneous behavior reflects individual variation rather than estrous state. Curr Biol.

